# Classification of Diabetic Retinopathy Severity in Fundus Images Using the Vision Transformer and Residual Attention

**DOI:** 10.1155/2023/1305583

**Published:** 2023-01-03

**Authors:** Zongyun Gu, Yan Li, Zijian Wang, Junling Kan, Jianhua Shu, Qing Wang

**Affiliations:** ^1^College of Medical Information Engineering, Anhui University of Traditional Chinese Medicine, Hefei 230012, China; ^2^Artificial Intelligence Research Institute of Hefei Comprehensive National Science Center (Anhui Artificial Intelligence Laboratory), Hefei 230026, China; ^3^Joint Surgery Department, Hefei First People's Hospital, Hefei 230061, China

## Abstract

Diabetic retinopathy (DR) is a common retinal vascular disease, which can cause severe visual impairment. It is of great clinical significance to use fundus images for intelligent diagnosis of DR. In this paper, an intelligent DR classification model of fundus images is proposed. This method can detect all the five stages of DR, including of no DR, mild, moderate, severe, and proliferative. This model is composed of two key modules. FEB, feature extraction block, is mainly used for feature extraction of fundus images, and GPB, grading prediction block, is used to classify the five stages of DR. The transformer in the FEB has more fine-grained attention that can pay more attention to retinal hemorrhage and exudate areas. The residual attention in the GPB can effectively capture different spatial regions occupied by different classes of objects. Comprehensive experiments on DDR datasets well demonstrate the superiority of our method, and compared with the benchmark method, our method has achieved competitive performance.

## 1. Introduction

Diabetic retinopathy (DR) is an ocular complication caused by diabetes. It is a leading cause of visual impairment and even blindness. It has become a major medical problem worldwide [1, 2]. However, up to now, there is no effective treatment for this disease. Studies have shown that early diagnosis and timely treatment of diabetic retinopathy are helpful to prevent blindness. This goal can be achieved through regular screening programs [3]. As a result, many national health agencies are promoting DR screening, which is effective in reducing blindness due to DR [4]. Digital fundus images are the most widely used imaging mode for ophthalmologists to screen and identify the severity of DR, and it can show the severity of the disease. However, due to the lack of ophthalmologists, DR screening is a heavy burden for many underdeveloped countries. For this reason, automatic classification technology for DR severity has become a trend in diagnosis.

With the development and application of artificial intelligence technology, deep learning [5] is playing a more and more important role in the field of medical image analysis. In recent years, the convolutional neural network (CNN) has been successfully applied to medical image classification [6, 7], medical image segmentation [8, 9], medical image registration [10, 11], medical image fusion [12, 13], and medical image report generation [14, 15] because it can learn highly complicated representations in a data-driven way. Although CNN shows great potential in medical image analysis, it also has some limitations. Local receptive fields such as convolution operations limit the capture of long-range pixel relationships. Inspired by the success of transformers in NLP, Alexey Dosovitskiy et al. [16] proposed the vision transformer (ViT), which takes image classification as a sequence prediction task for image patch sequences, to capture the long-range correlation of input image. In addition, recent research shows that compared with CNN, ViT is more in line with the prediction error of mankind [17, 18].

The biggest challenge of DR severity classification is that the classification accuracy of fundus disease images is more precise than other image categories. It shows that the differences of DR lesion points between main adjacent classes are very subtle, and it is difficult to distinguish. Although the attention module in ViT plays an important role in object classification, if the attention modules are simply superimposed, the performance of the model will decrease. In addition, ViT ignores the different spatial regions occupied by different kinds of objects. Motivated by the previously mentioned observations, we propose a deep network model to identify and classify DR, which consists of two key modules: FEB and GPB. The FEB module extracts the features of the image by the ViT model. The token in the ViT model has more fine-grained attention and pays more attention to the retinal hemorrhage area. The GPB module effectively captures the different spatial regions occupied by objects from different classes and generates class-specific features for each class by referring to a simple spatial attention score. By integrating the previous modules, our network can more accurately classify DR lesions of the different degrees.

To sum up, our contributions are as follows:

- Extracting fundus image features via vision transformer's excellent modeling ability.
- Using the residual attention module to make use of the individual spatial attention of each object class so as to improve the accuracy of DR classification.
- Experiments on DDR datasets show that this method has achieved good results in DR classification tasks. Specifically, our method achieves the best performance on grading 0, 2, 3, and 4.

## 2. Related Works

### 2.1. Pathological Analysis of the DR Severity

DR classification refers to the classification of retinal fundus images according to the severity of DR. In the fundus image, the bright region where the blood vessels converge is the optic disc, while the dark region on the other side is the fovea. Mild DR appears as dark red dots, which are small hemorrhages or small red dot-like microaneurysms. Moderate DR is the addition of some yellow lesions to the small red lesions, and some yellow-white punctate hard exudates appear. Severe DR adds some white cotton-like soft exudates to the red and yellow lesions, which have various types of shapes, from small spots to large plaques. The more such lesions, the more serious DR will be. Proliferative DR refers to the formation of new retinal blood vessels in or around the optic disc. It can cause vitreous hemorrhage and retina hemorrhage, and in severe cases, it can lead to retinal detachment. All these changes will be reflected in the fundus image.

**Figure 1:**
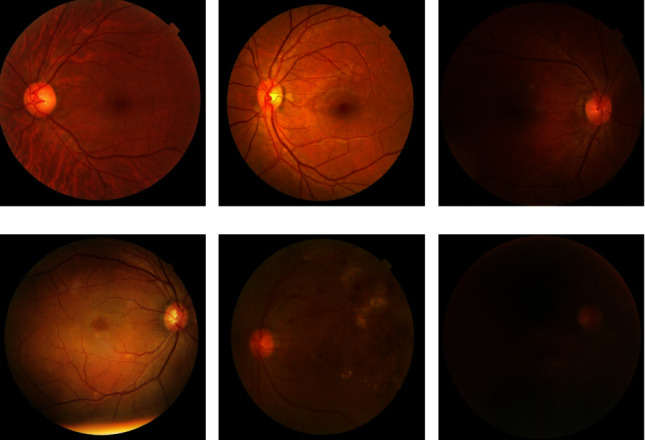
Example fundus images from the DDR dataset. (a) Healthy, which is labelled as class 0; (b) mild, which is labelled as class 1; (c) moderate, which is labelled as class 2; (d) severe, which is labelled as class 3; (e) proliferative, which is labelled as class 4; (f) ungradable, which is not up to quality standards to be used for model training and is labelled as class 5.

According to the international classification of DR [19], DR can be divided into five stages. They are class 0 (no DR), class 1 (mild DR), class 2 (moderate DR), class 3 (severe DR), and class 4 (proliferative DR). Figures 1(a)–1(e) show the five stages of DR, respectively [20]. As we all know, the image quality has a great influence on deep learning models. However, in clinical practice, due to exposure and other reasons, low-quality images are inevitable. Therefore, as shown in Figure 1(f), the DDR dataset [20] divided the fundus images that do not meet the quality standard into class 5 (ungradable).

### 2.2. Deep Learning in Medicine Images

With the rapid development of artificial intelligence (AI) technology, deep learning (DL) methods have been widely used in various tasks related to medical images and have achieved remarkable results. In the medical field, the types of images to be processed usually include X-ray, ultrasound, computed tomography (CT), and magnetic resonance imaging (MRI) [21, 22]. The processing tasks include image classification, object recognition, image segmentation, image reconstruction, and so on.

Medical image classification can assist doctors in diagnosing diseases. Esteva et al. [23] directly used 130,000 clinical image data to train the model based on the Inception v3 backbone network. The results showed that it was better than human experts. At the same time, the experiment proves that ordinary CNN can also produce good prediction results on large-scale and high-quality annotation data sets. Yi et al. [24] proposed a novel graph regularized NMF algorithm called NMF-LCAG that handles the adaptive graph learning issue in NMF. Compared with other related algorithms, the accuracy of the NMF-LCAG algorithm can be improved by at least 1%∼3% in most cases. To achieve efficient and rapid diagnosis of patients with COVID-19, Li et al. [25] proposed a computer-aided diagnosis algorithm based on ensemble deep learning. The experimental results show that the algorithm has good classification performance for COVID-19's disease patients, common pneumonia patients, and normal control groups and can significantly improve the performance of deep neural networks in multiclass prediction tasks.

It is of great significance in clinical treatment to accurately detect or identify lesions in medical images. The object recognition task is divided into two stages and one stage. The two-stage algorithm is represented by the R-CNN series [26–28], and the one-stage algorithm is most representative of the YOLO series [29–32]. Andrew Ng's group proposed the CheXnet algorithm [33], which is a 121-layer convolutional neural network. This algorithm can automatically detect pneumonia from chest X-rays. The accuracy rate is even higher than that of radiologists. Aoki et al. [34] completed the detection and probability prediction of erosion and ulcer lesions in wireless capsule endoscopy based on CNN for the first time. The detection accuracy reached 88.2%. In addition, properly improving the sensitivity of the model in clinical application will help doctors to reduce the missed detection rate.

The purpose of the medical image segmentation is to provide a reliable basis for clinical diagnosis and pathological research. The fully convolutional network (FCN) [35] was first used for segmented tasks. Although the final output layer has correct semantics, it is short of detailed information. U-Net [36] borrowed the idea of FCN, designed a more elegant image segmentation framework, and realized richer and more detailed segmentation results. In order to achieve precise segmentation of retinal blood vessels, Guo et al. [37] proposed a lightweight network named SA-Unet that achieves state-of-the-art performance on DRIVE and CHASE_DB1 datasets.

In addition, DL is also widely used in medical image reconstruction [38, 39], medical image report generation [40], and other tasks. Deep learning provides important theoretical basis and technical support for intelligent medicine. However, there are still many problems with intelligent medical imaging. For example, the lack of high-quality labelled training samples and the model obtained by deep learning is poorly interpretable.

### 2.3. Deep Learning in DR Classification

Accurate classification of medical images is an important means to assist clinical care and treatment. In recent years, DL has made remarkable achievements in medical image analysis, making DR-assisted diagnosis more reliable and efficient.

Bravo and Arbelez Pablo [41] investigated the performance of different preprocessing methods, designed a classification model based on the VGG16 architecture, and achieved an average classification accuracy of 50.5% in DR classification. A multi-cell architecture [42], which can gradually increase the depth of the deep neural network and the input image resolution, improves classification accuracy while reducing training time. To fully utilize images in different stages of deep learning, they also propose a multitask learning strategy. To solve the problem of lack of data, a deep learning architecture was proposed in [43], and the MESSIDOR dataset was used to train and test their architecture. Methods by developing the convolution layer and maximum pool layer in the first eight layers and the full connection layer in the last three layers, the AlexNet architecture is simply modified. The model is suitable for smaller datasets and provides acceptable accuracy. Golub et al. [44] put forward a method to identify and classify DR. This method could not only segment any retinal region of the fundus image but also evaluate the quality of the original image. In order to simulate the diagnosis process, a double-stream binocular network is proposed in [45] to capture subtle correlations between the left eye and the right eye, and its advantages over monocular methods are demonstrated on the EyePACS dataset. Zhang et al. [46] designed source-free transfer learning (SFTL) for DR detection, which utilizes unannotated retinal images and only employs a source model throughout the training process. On the EyePACS dataset, it achieved 91.2% accuracy, 0.951 sensitivity, and 0.858 specificity. [47] discusses existing DR detection and classification techniques, their advantages and disadvantages, and available DR datasets. The research achievements and progress in the field of DR detection are introduced in detail.

Although all these algorithms are devoted to extracting the features of lesions, there is still a problem of insufficient recognition performance of lesion, especially for small lesions. There are several reasons: (1) only high-resolution images can detect small pathological tissues, so the resolution of retinal images is very high. (2) Compared with other types of image, the classification accuracy of DR lesions is more accurate. Moreover, too small lesion points make the differences of DR lesion points between adjacent classes very subtle, which make it difficult to distinguish. (3) Identifying a severe class with a large local receptive field may lead to gradient disappearance or explosion problems. (4) The calculation cost of DR images is high, and it is difficult to train the model.

### 2.4. Visual Transformer in Medicine Images

Following the unprecedented success in natural language tasks, transformers [48] have also made great achievements in image recognition tasks recently. The ViT model has become very popular in various computer vision tasks including image classification [16], image detection [49], image segmentation [50], and so on. In the field of natural image recognition, ViT and its derived instances have achieved state-of-the-art performance on several benchmark datasets.

Recently, the ViT has been successfully applied in medical image classification. Yu et al. [51] proposed the MIL-ViT model, which was first pretrained on a large dataset of fundus images, and then fine-tuned on the downstream task of retinal disease classification. MIL-based headers are used in the MIL-ViT system and can be used with ViT in a plug-and-play way. Experiments on APTOS2019 and RFMiD2020 datasets show that the performance of MIL-ViT is better than that of the baselines based on CNN. Most data-driven methods regard DR classification and lesion detection as two independent tasks, which may not be optimal, because errors may be propagated from one stage to the next. To handle these two tasks together, it is proposed in [52] to the lesion aware transformer (LAT), which consists of an encoder-based pixel relationship and a decoder of the lesion-aware transformer. In particular, they take advantage of the transformer decoder to express lesion detection as a weakly supervised lesion localization problem. The LAT model has achieved the performance of the state of the art on the Messidor-1, Messidor-2, and EyePACS datasets. Yang et al. [53] proposed a hybrid structure consisting of the convolutional layer and the transformer layer to classify fundus diseases on OIA datasets. Similarly, Wu et al. [54] and AlDahoul et al. [55] also verified that the ViT model is more accurate than the CNN model in DR classification. As can be seen from the previous references, most methods directly use the original ViT model as a plug-and-play way to improve classification performance. Based on the previous observations, we think that using ViT as the backbone network to integrate domain-specific contexts can improve DR classification performance.

Apart from medical image classification, ViT is widely used in medical image segmentation [56], medical image detection [57], medical image reconstruction [58], medical image synthesis [59], and medical image report generation [60] and other tasks. However, some studies [61] have shown that the transformer is highly dependent on massive data, and its performance can surpass CNN only after training on large datasets. Most medical images have small public datasets and few labels, which limit the application of transformers in this field.

## 3. Methods

In this section, we first briefly outline our proposed network and then explain the key network components of the network in detail. Finally, the loss function of the designed is given.

### 3.1. Overview

**Figure 2:**
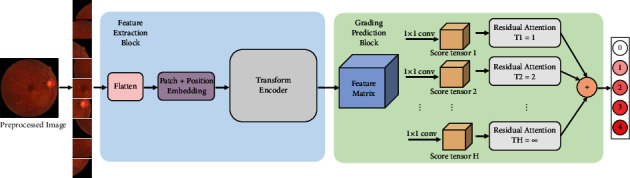
Pipeline of the proposed method.

Classification is usually based on differences or distinctions between categories. Diabetic retinopathy develops from mild to severe, and there is correlation between adjacent classes. For example, the severe DR stage follows the moderate DR stage, and the moderate DR stage follows the mild DR stage. Keeping this in mind, we propose a classification model that distinguishes five classes of DR through the ability of the ViT network to capture subtle changes and the discriminative ability of CSRA's different-category features. The model outputs five probability scores (which sum to 1), corresponding to the five classes of DR. The DR classification network architecture we designed is shown in Figure 2. It is composed of two key modules, FEB and GPB. FEB is mainly used for image feature extraction, while GPB is mainly used for classification prediction.

The proposed approach is presented as an algorithm in Algorithm 1.

### 3.2. Feature Extraction Block

As mentioned previously, the FEB is mainly used for extracting features from images. The standard transformer accepts 1-D token embedded sequences as input. To process a 2D image, we reshape the image *x* with the original shape [*H* × *W* × *C*] into a sequence of flattened 2D patches *x*_*P*_ ∈ *ℝ*^*N*×(*P*^2^*C*)^, where (*H*, *W*) represents the resolution of the original image, *C* represents the number of channels, (*P*, *P*) represents the resolution of each patch, and *N*=*HW*/*P*^2^ represents the number of patches. The transformer uses the same vector dimension *D* for all its layers, so we use a linear mapping layer to map the image patches to the *D* dimension. Similar to the [class] character in the BERT model, learnable embedding *z*_0_^0^=*x*_class_ is added before the block embedding sequence. The output state embedded in the transformer encoder is treated as an image representation. The processing of this process is shown in the following equation:

(1) $$\begin{array}{c} z_{0}=\left[x_{class};x_{p}^{1}E;x_{p}^{2}E;\cdots ;x_{p}^{N}E\right]+E_{pos}\text{,}\\ E\in R^{\left(P^{2}C\right)\times D},E_{pos}\in R^{\left(N+1\right)\times D}\text{,} \end{array}$$

where *E* is the weight vector of the linear mapping layer and *E*_pos_ is the positional embedding, which is directly added to the image patch embedding. The purpose of position embedding is to preserve the position information of different blocks, and the resulting sequence of embedded vectors is used as the input of the transformer encoder.

**Figure 3:**
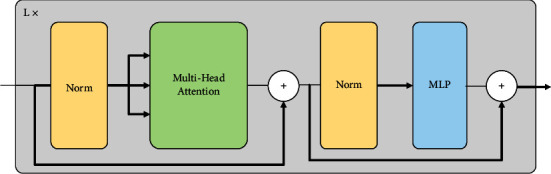
Transform encoder.

Figure 3 shows the network structure of the transformer encoder [16]. It consists of a stack of six identical encoding layers, each of which has two encoding sublayers. The first encoding sublayer is a multihead attention layer, while the second encoding sublayer is a position-wise feed-forward network. Residual connection is used between the two encoding sublayers, and the output of each encoding sublayer is normalized by the Layer Norm. Therefore, each sublayer can be represented as LN(Sublayer(*x*)), where Sublayer(*x*) is a function of the sublayer itself. For the convenience of residual connection, the output dimensions of all sublayers in the model are *d*_mode l_=512. In the transformer, the attention function maps the queries, keys, and value vectors into an output vector, which is packaged into matrices *Q*, *K,* and *V*, respectively. Attention is described in the following equation:

(2) $$\begin{array}{c} Attention\left(Q,K,V\right)=softmax\left(\frac{QK^{T}}{\sqrt{d_{k}}}\right)V\text{.} \end{array}$$

The multihead attention mechanism performs *L* different learnable linear mappings on the queries, keys, and value vectors and maps them into vectors of dimensions *d*_*k*_, *d*_*k*_, and *d*_*v*_, respectively. The head and multihead are described as follows:

(3) $$\begin{array}{c} {head}_{i}=Attention\left(QW_{i}^{Q},KW_{i}^{K},VW_{i}^{V}\right)\text{.} \end{array}$$

(4) $$\begin{array}{c} MultiHead\left(Q,K,V\right)=Concat\left({head}_{1},\ldots ,{head}_{h}\right)W^{O}\text{,} \end{array}$$

where the parameter matrix *W*_*i*_^*Q*^ ∈ *ℝ*^*d*_mode l_×*d*_*k*_^, *W*_*i*_^*K*^ ∈ *ℝ*^*d*_mode l_×*d*_*k*_^, *W*_*i*_^*V*^ ∈ *ℝ*^*d*_mode l_×*d*_*v*_^, and *W*^*O*^ ∈ *ℝ*^*hd*_*v*_×*d*_mode l_^.

### 3.3. Grading Prediction Block

Fundus image classification is a challenging computer vision task for practical applications. To capture the different spatial regions occupied by objects from different classes more efficiently, we introduce a class-specific residual attention algorithm [62] in GPB. With spatial attention scoring, the class-specific residual attention (CSRA) generates specific features for each class and then uses average pooling on these features for feature fusion.

**Figure 4:**
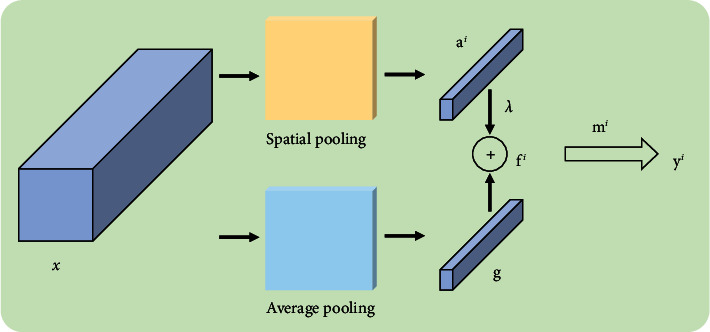
CSRA model.

As shown in Figure 4, the feature matrix *x* ∈ *ℝ*^*d*×*h*×*w*^ of the input image is extracted by the FEB. Here, *d*, *h,* and *W* represent the dimension, height, and width of the feature matrix, respectively, and we assume that *d* is 2048, *h* is 7, and *W* is 7. Firstly, the feature matrix *x* is decoupled into a position feature matrix group *x*_1_, *x*_2_,…, *x*_49_ (*x*_*j*_ ∈ *ℝ*^2048^). Then, a fully connected layer (1 × 1 convolution) is used as the classifier. Note that each class has its own specific fully connected layer classifier, and the parameter of the classifier *m*_*i*_ corresponding to the *i*th class is *m*_*i*_ ∈ *ℝ*^2048^.

The CSRA score is defined in [62] by the following equation:

(5) $$\begin{array}{c} s_{j}^{i}=\frac{\exp\;\left(Tx_{j}^{T}m_{i}\right)}{\overset{49}{\underset{k=1}{\sum }}\exp\left(Tx_{k}^{T}m_{i}\right)}\text{,} \end{array}$$

where *T* (>0) is the temperature control factor and *s*_*j*_^*i*^ is the probability that class *i* appears at the position *j*.

The CSRA *f*^*i*^ for the class *i* is given by the following equation:

(6) $$\begin{array}{c} f^{i}=g+\lambda a^{i}\text{.} \end{array}$$

Here, *a*^*i*^ is a class-specific feature vector and a^*i*^=∑_*k*=1_^49^*s*_*k*_^*i*^x_*k*_. *λ*is a hyperparameter (setting *λ* = 0.3), g=1/49∑_*k*=1_^49^x_*k*_.

According to [62], the dot product of the CSRA *f*^*i*^ of the *i*th class and the classifier *m*_*i*_ corresponding to this class obtain the final logical output, as shown in the following equation:

(7) $$\begin{array}{c} \hat{y}≜\left(y^{1},y^{2},\ldots ,y^{C}\right)=\left({m_{1}^{T}f}^{1},{m_{2}^{T}f}^{2},\ldots ,{m_{C}^{T}f}^{C}\right)\text{.} \end{array}$$

Here, *C* is the number of classification categories.

### 3.4. Loss Function

In this paper, the binary cross-entropy (BCE) loss function given in (8) is used to calculate the loss between prediction *y* and label $\hat{y}$ of ground truth. The stochastic gradient descent (SGD) method is used to optimize the loss function.

(8) $$\begin{array}{c} L=-\overset{N}{\underset{i=1}{\sum }}y_{i}\log{\hat{y}}_{i}+\left(1-y_{i}\right)\log\;\left(1-{\hat{y}}_{i}\right). \end{array}$$

## 4. Experiments and Results' Discussion

### 4.1. Datasets

The DDR dataset is provided by Ocular Disease Intelligent Recognition (ODIR-2019) for lesion segmentation and lesion detection [20]. This dataset consists of 13,673 fundus images from 147 hospitals, covering 23 provinces in China. For DR classification tasks, the division of the training set, validation set, and test set is provided on DDR, of which 6835 are used for training, 2733 are used for validation, and the remaining 4105 are used for testing. The DR images in DDR are divided into six classes: no DR, mild nonproliferative DR, moderate nonproliferative DR, severe nonproliferative DR, proliferative DR, and ungradable.

The IDRiD dataset (The Indian Diabetic Retinopathy Image Dataset) is the first database representing the Indian population [63]. It is a dataset consisting of typical DR and normal retinal structures and is divided into three parts, namely, segmentation, classification, and location. Among them, classification consists of 516 original color fundus images which are divided into the train set (413 images) and test set (103 images). In addition, this dataset provides information on the disease severity of DR and diabetic macular edema for each image. This makes it ideal for the development and evaluation of image analysis algorithms for the early detection of DR.

### 4.2. Implementation Details

In order to prepare more trainable data, we do some operations on the original images. In this paper, the pretrained backbone model parameters are used, and the training is fine-tuned on the used datasets. Limited by the memory received, the large images are randomly resized 512 × 512 sizes. In addition, we apply random horizontal flips, vertical flips, and random rotation as forms of data augmentation to reduce overfitting. Our framework is implemented by PyTorch 1.6 and runs on NVIDIA Quadro RTX 6000 GPU with 24 GB of memory.  Table 1 highlights the hyperparameters used in training.

### 4.3. Evaluation Metrics

We select the following indicators to evaluate the performance of the classification model. These indicators are calculated according to equations (9)–(13): (9) precision, which means the proportion of samples that are correctly classified as positive; (10) recall/sensitivity, which is the probability that the DR image of the lesion is not missed as negative; (11) specificity, which is the probability that a DR image with normal specificity will not be misjudged as positive; (12) accuracy represents the correct proportion of model classification; (13) *F*1-score, which is the harmonic mean between precision and recall. These initial metrics are added to a confusion matrix of multiclassification. Equations (14)–(17) make it possible to extend the definitions of these performance indicators to *N* classes. This work uses the following indicators (macro-average): accuracy, sensitivity, specificity, and *F*1-score to evaluate the DR classification process.

(9) $$\begin{array}{c} Precision=\frac{TP}{TP+FP\;}\text{,} \end{array}$$

(10) $$\begin{array}{c} Recall=\frac{TP}{TP+FN\;}\text{,} \end{array}$$

(11) $$\begin{array}{c} Specificity=\frac{TN}{TN+FP\;}\text{,} \end{array}$$

(12) $$\begin{array}{c} Accuracy=\frac{TP+TN}{TP+FP+FN+TN\;}\text{,} \end{array}$$

(13) $$\begin{array}{c} F1-score=\frac{2\;*\;Precision\;*\;Recall}{\left(Precision+Recall\right)}\text{,} \end{array}$$

(14) $$\begin{array}{c} TP\;for\;class\;k=A_{k,k}\text{,} \end{array}$$

(15) $$\begin{array}{c} FP\;for\;class\;k={\left(\sum_{i=0}^{n}A_{i,k}\right)-A}_{k,k}\text{,} \end{array}$$

(16) $$\begin{array}{c} FN\;for\;class\;k={\left(\sum_{i=0}^{n}A_{k,i}\right)-A}_{k,k}\text{,} \end{array}$$

(17) $$\begin{array}{c} TN\;for\;class\;k={\left(\sum_{i=0}^{n}\sum_{j=0}^{n}A_{i,j}\right)-\sum_{i=0}^{n}A_{i,k}-\sum_{i=0}^{n}A_{k,i}+A}_{k,k}\text{,} \end{array}$$

where TP (true positive) represents the positive samples predicted by the model to be in a positive class, TN (true negative) represents the negative samples predicted by the model to be in the negative class, FP (false positive) represents the negative samples predicted to be positive by the model, and FN (false negative) indicates the positive sample predicted by the model as a negative class.

In addition, the area under the curve (AUC) of the receiving operating characteristic (ROC) curve is employed, which is also recognized as a metric of fundus image grading in previous research. The AUC reflects the performance of the data predicted positively and also characterizes the effectiveness of the model. The higher the AUC value, the better the effect of model classification.

### 4.4. Evaluation of the Model Performance

**Figure 5:**
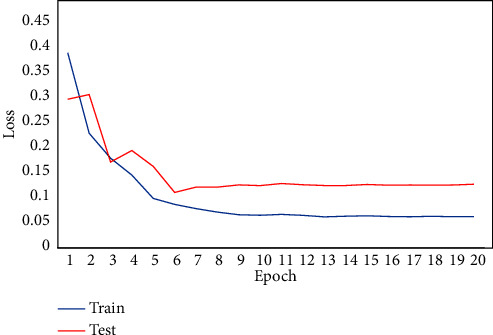
Loss curves on the train set and test of DDR.

In this work, the DR classification method in 5 severity categories from 0 to 4 is proposed. An additional category (class 5), similar to [20], is related to images presenting artifacts, which prevent clear evaluation of information generated. A second experiment was performed to exclude images with artifacts (class 5). To evaluate the performance of our model, we trained and tested it on DDR and IDRiD datasets.  Figure 5 is the loss curve of our model training process on the DDR dataset. It can be seen that the loss decreases with the increase in training times. In the first 6 epochs, the loss value of the test set and the train set decreases significantly. The loss value of the test set does not decrease after the 7th epoch and the train set after the 9th epoch, and the model training tends to be saturated. From the change of the loss curves, it can be seen that there is little difference between the train loss and the test loss, which means that our model does not show overfitting.

For the prediction classification model, we hope that the more accurate the prediction results of the model, the better. That is, the larger the value of TP and TN in the confusion matrix, the better and the smaller the value of corresponding FP and FN. However, confusing the matrix will calculate the number. With a large amount of data, it is difficult to directly judge whether the model is good or bad. Besides, accuracy is not a good indicator for unbalanced data sets. Therefore, on the basic statistical results of the confusion matrix, we introduced five metrics: precision, recall, specificity, *F*1-score, and accuracy.

The six classification confusion matrices and the five classification confusion matrices obtained by this method are shown in Tables 2 and 3, respectively. From the two tables, it can be seen that although there are misclassifications between classes, most of them are classified into adjacent classes. Most of the data fall on the diagonal line. In addition, most of the data fall on the diagonal line, which also proves that this algorithm is suitable for DR image classification.

Tables 4 and 5 present the metrics obtained from this work, separated by class and database. It was observed that in both results, in cases with no DR and proliferative stage classes, the DR model has a high classification index. It can be analyzed that the model can distinguish the categories with distinct characteristics. In the intermediate classes (from 1 to 3), the classification index of the DR model is not high. This is because there are no obvious differences between the characteristics of these categories, and it is easy to be confused with nearby categories. Comparing the results in Tables 4 and 5, the results obtained show that including ungradable category (class 5) improves accuracy in all categories. This also reflects that the image quality plays an important role in the classification of the model.

**Figure 6:**
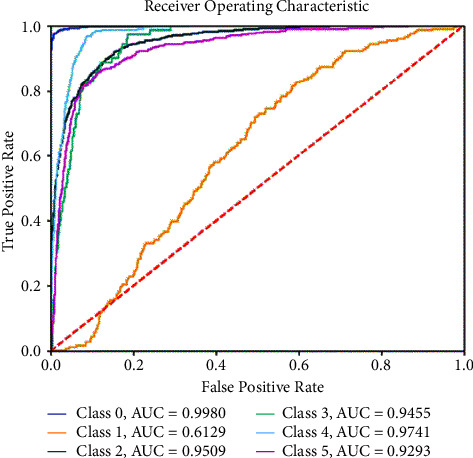
The ROC and AUC per category for DR classification on the DDR dataset.

The ROC curve and AUC value are used to evaluate the performance of our model. As shown in Figure 6, the AUC values of class 0, class 1, class 2, class 3, class 4, and class 5 were 0.9980, 0.6129, 0.9509, 0.9455, 0.9741, and 0.9293, respectively. Our model performed well enough in class 0, class 2, class 3, class 4, and class 5. However, the performance of class 1 was not satisfactory. In addition to the possible reasons we have analyzed previously, the serious imbalance of the amount of data is also a very important factor. After all, the total sample size in the DDR dataset is 13673, while the sample size of class 1 is only 630. To solve this problem, we tried to resample the data and retrain the model by binary classification. The AUC of class 1 can reach 0.9430. In this way, if it is just a simple DR screening, the binary classification model can be used in clinical applications for mild patients. If you want to evaluate the risk level of patients and image quality, you can use the multiclass model.

### 4.5. Comparing with Other Methods

Comparing the accuracy and average accuracy (AA) of our method with previous work reported on the DDR dataset (Table 6), our method ranks first in class 1, class 2, class 3, class 4, class 5, and AA. Our model achieved an accuracy of 0.9635 on class 1. The state-of-the-art performance is achieved among all models, which is almost 4 times the accuracy of the second-best DenseNet-121 model (0.2275). This shows that our model has made great improvement on the most difficult part to identify mild DR, and our model has also achieved the best performance in class 2, class 3, and class 4. The performance has greatly improved. For the purposes of image quality control, our model has also been improved by 3.30% compared with the second-best VGG-16 model. For the AA metric, our model achieved a performance of 0.9154, which was 30.35% higher than the second-best DenseNet-121 model. However, our model does not perform as well as other models at level 0. Our model is not set manually, but the optimal threshold is obtained according to the Youden index. It can be speculated that the reason is that the model made concessions in order to reduce the rate of missed detection and improve overall performance. In conclusion, compared with other benchmark models, the model which is based on ViT and CSRA is highly competitive in DR severity classification.

### 4.6. Ablation Studies on the DDR Dataset

In this paper, the influence of each component in the network is studied by an ablation experiment. First, we replace the transformer with a different backbone to verify the influence of FEB on our model. Then, CSRA is replaced by MLP, and there was no change in the FEB module, to verify the influence of GPB on our model. At last, on the basis of keeping the existing model unchanged, we set the different number of heads of CSRA in GPB to verify the influence of CSRA parameters setting on the overall performance of the model. Tables 7 and 8 detail the sensitivity, specificity, accuracy, and AUC values obtained from different experiments and can be compared.

1. Analyze the effect of FEB: first of all, the FEB part takes ResNet50 as the backbone to extract image features. Compared to this design, our model improves sensitivity by nearly 2%, specificity by nearly 4%, accuracy by nearly 3%, and AUC by more than 6%. Then, the FEA part uses ResNet101 as the backbone of extracting image features. Compared with this design, the sensitivity and specificity of our model are only slightly improved, and the accuracy and average AUC values are increased by over 1%, respectively.
2. Analyze the effect of GPB: next, we keep the FEB module unchanged and replace it with MLP by CSRA. Compared with this design, the sensitivity and accuracy of our model are improved by more than 2%, the specificity by more than 4%, and the AUC value by nearly 4%, respectively. As can be seen from Table 8, our model has achieved the best performance in all the evaluation indexes.
3. Analyze the effect of attention heads in GPB: according to the research results of [62], we set the head to 2 by default. In order to verify the influence of attention heads on our model and keep the existing model unchanged, the number of CSRA heads is set to 1, 4, and 6, respectively. It can be seen from the experimental results in Table 8 that our parameter setting achieves the best performance (Head = 2).

## 5. Conclusions

According to the data of the International Diabetes Federation, diabetes is one of the fastest-growing global health emergencies in the 21st century. By 2030, it is estimated that 643 million people will have diabetes (accounting for about 11.3% of the global population) [1]. DR is one of the common chronic complications of diabetes. Due to the different stage of DR severity, it can be divided into five stages from mild to severe. In this paper, we design a new network to classify the fundus images of DR different stages by using vision transformers and residual attention. The model is trained and tested on two publicly available fundus image datasets (DDR dataset and IDRiD dataset). The experimental results show that compared with the existing five DR classification benchmark methods, the proposed model has better performance. However, limited by the number of labelled samples and the imbalance of data, there is still a lot of room for improvement in the identification and classification of mild DR, which leads to the deficiency of our network. Therefore, in future work, we will continue to improve the network structure and further modify the learning strategy to achieve better classification performance of DR severity.

## Figures and Tables

**Algorithm 1:**
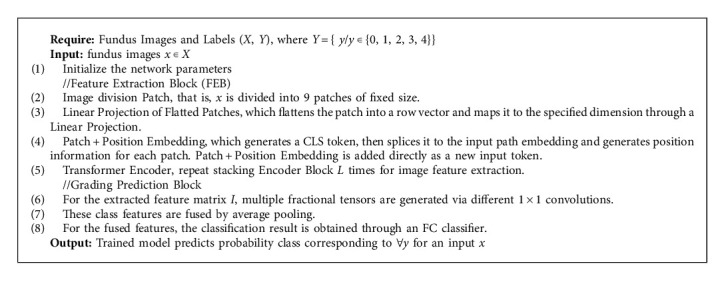
Training the classification of the DR model.

**Table 1:** The DR model training hyperparameters.

Hyperparameters	Valor
Optimizing function	SGD optimizer
Momentum	0.9
Weight decay	5 × 10^−4^
Epochs	20
Batch size	32
Initial learning rate	1 × 10^−3^
Dropout	0
Classifier	0.01
Number of classes	5 and 6 classes

**Table 2:** Confusion matrix on the DDR dataset for DR classification model.

Actual label	Predict label					
	0	1	2	3	4	5
0	**1847**	5	28	0	0	0
1	65	**79**	42	0	1	2
2	576	34	**639**	14	28	53
3	6	1	17	**38**	8	1
4	9	0	87	2	**152**	25
5	3	0	6	0	12	**325**

Bold font indicates the best result in each column.

**Table 3:** Confusion matrix on the DDR dataset for the IDRiD classification model.

Actual label	Predict label				
	0	1	2	3	4
0	**20**	0	14	0	0
1	2	**3**	0	0	0
2	3	0	**29**	0	0
3	0	0	9	**10**	0
4	1	0	2	0	**10**

Bold font indicates the best result in each column.

**Table 4:** Performance measures of the DR classification model for DDR.

Class	Precision	Recall	Specificity	*F*1-score	Accuracy
0	0.7329	0.9824	0.0208	0.8395	0.8280
1	0.6639	0.4180	0.0276	0.5130	0.9635
2	0.7607	0.4754	0.2159	0.5852	0.7793
3	0.7037	0.5352	0.0081	0.6080	0.9881
4	0.7562	0.5527	0.0315	0.6387	0.9581
5	0.8005	0.9393	0.0057	0.8644	0.9752

**Table 5:** Performance measures of the DR classification model for IDRiD.

Class	Precision	Recall	Specificity	*F*1-score	Accuracy
0	0.7692	0.5882	0.1818	0.6667	0.8058
1	1.0000	0.6000	0.0200	0.7500	0.9806
2	0.5370	0.9063	0.0612	0.6744	0.7282
3	1.0000	0.5263	0.0968	0.6897	0.9126
4	1.0000	0.7692	0.0323	0.8696	0.9709

**Table 6:** Performance comparison of different models on the DDR dataset.

Model	0	1	2	3	4	5	AA
VGG-16 [64]	0.9537	0.0423	0.5625	0.3944	*0.6436*	*0.9422*	0.5898
ResNet-18 [65]	*0.9548*	0.0582	0.622	0.3662	0.5818	0.9133	0.5827
GoogLeNet [66]	**0.9574**	0.0265	0.5759	0.3380	0.5782	0.9162	0.5654
DenseNet-121 [67]	0.8930	*0.2275*	0.5751	*0.4085*	0.6364	0.9306	*0.6119*
SE-BN-Inception [68]	0.9452	0.0476	*0.6458*	0.1268	0.5818	0.9046	0.5418
ViT + CSRA	0.8280	**0.9635**	**0.7793**	**0.9881**	**0.9581**	**0.9752**	**0.9154**

The bold font represents the best performance on that class. The italic font indicates the second-best performance on that class.

**Table 7:** The results of different ablation experiments in the DDR dataset. Sensitivity, specificity, accuracy, and AUC are average values, respectively.

Method	Sensitivity	Specificity	Accuracy	AUC
ResNet50 + CSRA	0.7941	0.7846	0.7940	0.8405
ResNet101+CSRA	*0.8120*	*0.8242*	*0.8130*	*0.8903*
ViT + MLP	0.7929	0.7815	0.7980	0.8647
ViT + CSRA	**0.8140**	**0.8245**	**0.8235**	**0.9018**

The bold font represents the best performance in each column. The italic font represents the second-best performance in each column.

**Table 8:** The effect of the number of attention heads in the DDR dataset. Similar to Table 7, here sensitivity, specificity, accuracy, and AUC are average values, respectively.

Head setting	Sensitivity	Specificity	Accuracy	AUC
Head = 1	0.8037	0.7925	0.7827	0.8436
Head = 4	*0.8064*	*0.8082*	*0.8241*	*0.8912*
Head = 6	0.7932	0.8030	0.8148	0.8643
Ours (head = 2)	**0.8140**	**0.8245**	**0.8235**	**0.9018**

The bold font represents the best performance in each column. The italic font represents the second-best performance in each column.

## Data Availability

The DDR and IDRiD datasets used to support the findings of this study are available from the corresponding author upon request.
